# Hemocyte Clusters Defined by scRNA-Seq in *Bombyx mori*: *In Silico* Analysis of Predicted Marker Genes and Implications for Potential Functional Roles

**DOI:** 10.3389/fimmu.2022.852702

**Published:** 2022-02-25

**Authors:** Min Feng, Luc Swevers, Jingchen Sun

**Affiliations:** ^1^ Guangdong Provincial Key Laboratory of Agro-animal Genomics and Molecular Breeding, College of Animal Science, South China Agricultural University, Guangzhou, China; ^2^ Insect Molecular Genetics and Biotechnology, Institute of Biosciences & Applications, National Centre for Scientific Research “Demokritos”, Aghia Paraskevi, Athens, Greece

**Keywords:** scRNA-seq, silkworm, hemocyte, marker gene, *Bombyx mori*

## Abstract

Within the hemolymph, insect hemocytes constitute a heterogeneous population of macrophage-like cells that play important roles in innate immunity, homeostasis and development. Classification of hemocytes in different subtypes by size, morphology and biochemical or immunological markers has been difficult and only in *Drosophila* extensive genetic analysis allowed the construction of a coherent picture of hemocyte differentiation from pro-hemocytes to granulocytes, crystal cells and plasmatocytes. However, the advent of high-throughput single cell technologies, such as single cell RNA sequencing (scRNA-seq), is bound to have a high impact on the study of hemocytes subtypes and their phenotypes in other insects for which a sophisticated genetic toolbox is not available. Instead of averaging gene expression across all cells as occurs in bulk-RNA-seq, scRNA-seq allows high-throughput and specific visualization of the differentiation status of individual cells. With scRNA-seq, interesting cell types can be identified in heterogeneous populations and direct analysis of rare cell types is possible. Next to its ability to profile the transcriptomes of individual cells in tissue samples, scRNA-seq can be used to propose marker genes that are characteristic of different hemocyte subtypes and predict their functions. In this perspective, the identities of the different marker genes that were identified by scRNA-seq analysis to define 13 distinct cell clusters of hemocytes in larvae of the silkworm, *Bombyx mori*, are discussed in detail. The analysis confirms the broad division of hemocytes in granulocytes, plasmatocytes, oenocytoids and perhaps spherulocytes but also reveals considerable complexity at the molecular level and highly specialized functions. In addition, predicted hemocyte marker genes in *Bombyx* generally show only limited convergence with the genes that are considered characteristic for hemocyte subtypes in *Drosophila*.

## 1 Introduction

The blood cells or hemocytes play an important role in the regulation of whole-body homeostasis in insects. While originally implicated in the cellular response against microorganisms and parasites, functions of hematocytes have continuously expanded to include tissue remodeling during development, wound repair, storage and distribution of nutrients and the humoral immune response (1, 2). In their motility and versatility to respond to various signals insect hemocytes resemble the myeloid-like blood cells in vertebrates (2, 3).

The diversity of hemocytes has been mostly studied in lepidopteran insects and in *Drosophila* (4–7). Despite the different nomenclature used, differentiated and functional hemocytes can be generally divided in three main types by morphological and functional criteria (8, 9).

The first category constitutes “macrophage-like cells” that are named plasmatocytes in *Drosophila* and correspond to granular hemocytes (granulocytes) in Lepidoptera (8). The main function of the macrophage-like cells is phagocytosis; while generally belonging to the same category, lepidopteran granulocytes differ from *Drosophila* plasmatocytes by the presence of specialized inclusions (granules) that are released upon activation (10, 11).

Cells that exhibit extensive spreading behavior and form capsules around large foreign bodies or nodules around bacteria and fungi are called lamellocytes in *Drosophila.* Confusingly, hemocytes with similar properties are called plasmatocytes in Lepidoptera (8). However, so-called plasmatocytes form a permanent part of the circulating hemocyte population in Lepidoptera (1), while lamellocytes are mainly observed in the context of parasitization in *Drosophila* (12), indicating functional differences.

Oenocytoids form a group of fragile cells in Lepidoptera that are characterized by the production of phenoloxidase that is responsible for melanization reactions in the hemolymph (1). In *Drosophila*, crystal cells show highly similar cytological and biochemical features (13) and therefore can be considered the equivalent of oenocytoids (8).

Other hemocyte types occur with lower abundance in the hemolymph. Pro-hemocytes have a round shape with a high nucleus-to-cytoplasm ratio and are considered the precursor cells of the other hemocyte types in both Lepidoptera and *Drosophila* (14, 15). Spherule cells or spherulocytes are recognized as another differentiated hemocyte cell type in Lepidoptera (with unknown function) but do not occur in *Drosophila* (16). Other rare specialized hemocyte cell types may appear in the hemolymph under specific conditions (8, 17).

## 2 Application of Single Cell RNA Sequencing for the Identification of Hemocyte Subsets

The technique of single cell RNA sequencing (scRNA-seq) has emerged as a powerful technique for the characterization of cell heterogeneity and the identification of individual cell types in tissues containing multiple types of cells (18, 19). After identification of cell groups by clustering approaches, differential expression analysis can be performed in conjunction with functional profiling for the identification of biomarkers that are characteristic for the detected cell groups (20). Construction of the cell atlas and the definition of corresponding marker genes are considered a valuable resource for the execution of follow-up functional studies in various biological processes (21).

The application of scRNA-seq on blood cells has the advantage of straightforward isolation of single cells that does not require prior digestion as is the case for solid tissues (22) and was used successfully to characterize hemocyte populations in the hemolymph of *Drosophila* larvae (4, 5, 23). Based on differential gene expression levels, 16 clusters or subpopulations were identified, which were found to broadly correspond to the hemocyte subtypes identified by morphological and physiological criteria: 12-13 clusters of (*Drosophila*) plasmatocytes, 1-2 clusters of crystal cells, and 2 clusters of lamellocytes (reported in two independent studies: 4, 5). Most notably, the assignment of clusters to belong to the three hemocyte types was guided using known markers such as *lozenge* for crystal cells, *NimC1* for plasmatocytes and *atilla* for lamellocytes (4, 5).

The 16 clusters/subpopulations of hemocytes in *Drosophila* larvae showed different abundances according to three experimental conditions: unwounded, wounded and parasitoid wasp-infected (5). As expected, the two clusters representing lamellocytes were absent in hemolymph of unwounded larvae (4). Another study employing scRNA-seq focused on the lymph gland or the larval hematopoietic organ of *Drosophila* and identified new hemocyte types including adipohemocytes and different subtypes of pro-hemocytes (23).

The technique of scRNA-seq was also applied to hemocytes of silkworm (*Bombyx mori*, Lepidoptera) (24). In this study, hemocytes were isolated from 5^th^ instar larvae at three days after experimental manipulation which consisted either of (1) injection into the body cavity of a high infectious dose of *B. mori* nucleopolyhedrovirus (BmNPV) (baculovirus infection) or (2) injection of saline solution (wounding). After normalization, gene expression data from 22,286 cells were subjected to dimensionality reduction methods such as component analysis (PCA), *t*-distributed stochastic neighbor embedding (*t*-SNE) and uniform manifold approximation and projection (UMAP) (25). The R package Seurat (26) was used as a graph-based clustering method to obtain 20 hemocyte cell type clusters and to screen for marker genes in each cluster (24).

The cluster analysis of scRNA-seq of *Bombyx* hemocytes revealed that cell clusters associated with baculovirus infection (7 clusters: 1, 2, 3, 9, 11, 13, 18) were clearly separated from the cell clusters of the (wounded) control (9 clusters: 0, 4, 5, 7, 8, 10, 12, 16, 17), which reflects the severe impact of baculovirus infection on the composition of the hemocyte population (24). In 4 clusters (6, 14, 15, 19), the contribution from both control and BmNPV-infected groups is less polarized (10-30% contributed by cells from BmNPV-infected group) (**Table 1**).

**Table 1:** General characteristics of hemocyte clusters identified by scRNA-seq in *Bombyx mori* larvae.

Cluster	Number of cells	Contribution by BmNPV-infected group (%)	Number of DEGs	Highest log2FC value	Hypothesized Specialized Function
**Clusters that are predominant in control group**					
0	3145	0.2	814	2.73	Phagocytosis (Intermediary)
4	1643	0.2	1310	4.33	Proliferation
5	1511	3.3	613	3.37	Stress response (Intermediary)
7	1270	0.3	340	3.08	Tissue repair (Intermediary)
8	1174	0.6	269	2.30	Pattern recognition
10	1026	1.0	1571	4.70	Tissue repair (Cecropin B)
12	832	0.3	260	6.35	Migration Tissue repair Stress response
16	184	0.0	366	3.91	Extracellular protease cascade (Coagulation, Melanization) Stress response
17	153	0.0	170	2.22	Tissue repair (Cecropin B)
**Clusters that are predominant in BmNPV-infected group**					
1	2315	99.6	108	1.18	(Baculovirus Infection)
2	2056	96.1	127	0.90	(Baculovirus Infection)
3	1745	99.7	180	2.04	(Baculovirus Infection)
9	1173	99.8	181	3.88	(Baculovirus Infection)
11	940	98.2	152	5.72	(Baculovirus Infection)
13	697	99.9	98	6.64	(Baculovirus Infection)
18	57	100.0	105	7.74	(Baculovirus Infection)
**Clusters with significant contribution from BmNPV-infected group**					
6	1363	11.4	1602	2.98	Protein synthesis
14	653	10.7	1734	9.72	Stress Response Encapsulation and melanization (Regulatory)
15	299	28.2	835	12.39	Encapsulation and melanization (Effector)
19	50	22.0	871	13.23	Tissue repair (Effector)

The contribution of cells from the control and BmNPV-infected groups is indicated. DEGs, differentially expressed genes. Among the clusters that mainly consist of uninfected cells, clusters 0, 4, 6, 7, 10 and 17 are considered as granulocytes (green; 8600 cells), clusters 5, 8, 12 and 16 may represent oenocytoids (blue; 3701 cells), clusters 14 and 15 are classified as plasmatocytes (red; 952 cells) while spherulocytes occur as the single cluster 19 (purple; 50 cells). Hemocyte clusters that are heavily infected with baculovirus (clusters 1, 2, 3, 9, 11, 13, 18) are proposed to consist of pro-hemocytes that have invaded the hemolymph from the hematopoietic organs as an antiviral defense mechanism (yellow; 8983 cells). With respect to uninfected cells, granulocytes constitute the highest proportion (65%), followed by oenocytoids (29%), plasmatocytes (6%) and spherulocytes (<0.5%). These proportions differ from those obtained by hemocyte counts in larvae based on morphology: granulocytes (59-69%), plasmatocytes + pro-hemocytes (18-24%), oenocytoids (4-9%) and spherulocytes (6-10%) (27), indicating overlap in morphological features among different subtypes defined by scRNA-seq. In Manduca sexta, the proportions are: granulocytes (67%), plasmatocytes (16%), pro-hemocytes (6%), oenocytoids (1%) and spherulocytes (10%) (28).

The cell clusters that are predominant in the BmNPV-infected group are characterized by much lower number of differentially expressed genes (DEGs) (range: 98 to 181) compared to the control group (range: 170 to 1571) (**Table 1**) and their morphological features resemble those of pro-hemocytes (24). It was therefore hypothesized that the BmNPV-infected cells in clusters 1, 2, 3, 9, 11, 13 and 18 represent pro-hemocytes that were released from the hematopoietic organs in *Bombyx* larvae to replenish the loss of differentiated hemocytes (granulocytes, plasmatocytes, oenocytoids) in the hemolymph following baculovirus infection (24).

For the (wounded) control group of hemocyte clusters in *B. mori*, attempts were made to assign the clusters to the classic morphological and cytochemical categories of plasmatocytes, granulocytes and oenocytoids of lepidopteran larvae (8). Based on markers found in the literature, clusters 5, 8, 12 and 16 were identified as oenocytoids (expressing *PPBP1* and *PPBP2*, encoding paralytic peptide-binding proteins; 16), clusters 14 and 15 were assigned as (lepidopteran) plasmatocytes (marker genes: *serine protease homolog1, β-1,3-glucan recognition protein 3, paralytic peptide* and *integrin β3*; 16, 29), and clusters 0, 4, 6, 7, 10, 17 were tentatively designated as granulocytes (based on the expression of *scavenger receptor-C, cathepsin B, integrin α3, hemocyte protease-1* and *peptidoglycan recognition protein precursor*; 16, 30). In addition, cluster 19 was labeled as spherulocyte-like based on the preferential expression of *cathepsin L-like* (16).

Nevertheless, the different lineages of hemocytes and their developmental pathways are much less well understood in *Bombyx* than in *Drosophila*, and the assignment of clusters to different hemocyte types in *Bombyx* can only be regarded as tentative and will require further validation. Concurrently, many new marker genes were predicted based on differential gene expression and functional profiling in the designated clusters, which were not discussed in detail in the first article that presented the definition of hemocyte clusters in *B. mori* larvae following scRNA-seq analysis (24). Recently, lectins and monoclonal antibodies have allowed a more robust classification of hemocytes types in lepidopteran insects (7, 31) but the identity and the function of the marker molecules that are detected largely remain unknown. Sequences of marker genes assigned by scRNA-seq to specific clusters, on the other hand, contain sufficient information to allow the prediction of their function based on knowledge from better studied model organisms, most notably *Drosophila*, for which scRNA-seq data of hemocyte types are already available (4, 5, 23). In the analysis that follows, an attempt is made to predict the function of the hemocyte types in *B. mori* larvae based on the marker genes that were proposed following scRNA-seq analysis.

## 3 Methodological Approach

In the previous article (24), a likelihood-ratio test (32), performed on single cluster cells against all other cells, was used to identify DEGs in single silkworm hemocyte clusters based on differential expression. Up-regulated DEGs in each cluster were identified by the following criteria: 1) p value ≤ 0.01; 2) log2 fold change (log2FC) ≥ 0.360674 (log2FC means log fold change of the average expression between the two groups); and 3) percentage of cells in a specific cluster where the gene is detected > 25%. The top genes in each silkworm hemocyte cluster were then selected as the potential marker genes for each cluster.

To infer the function of proposed marker genes, a literature search was performed with as key word the name of the gene in association with terms such as “hemocyte” and “innate immunity” and species or taxon names such as “*Drosophila*”, “*Bombyx mori*”, “Lepidoptera” and “insects”. If no gene name was provided on the database SilkDB 3.0 (33), protein sequences were subjected to BLAST searches as well as the HHpred interactive server for remote protein homology detection and structure prediction (34). The predicted function of the 5-6 top marker genes was used to construct the main activity of the cells in the identified clusters.

The datasets analyzed in this study can be found in online repositories at https://www.ncbi.nlm.nih.gov/bioproject/?term=PRJNA658439.

## 4 *In Silico* Analysis of Predicted Marker Genes for Each Cluster

**Figure 1:**
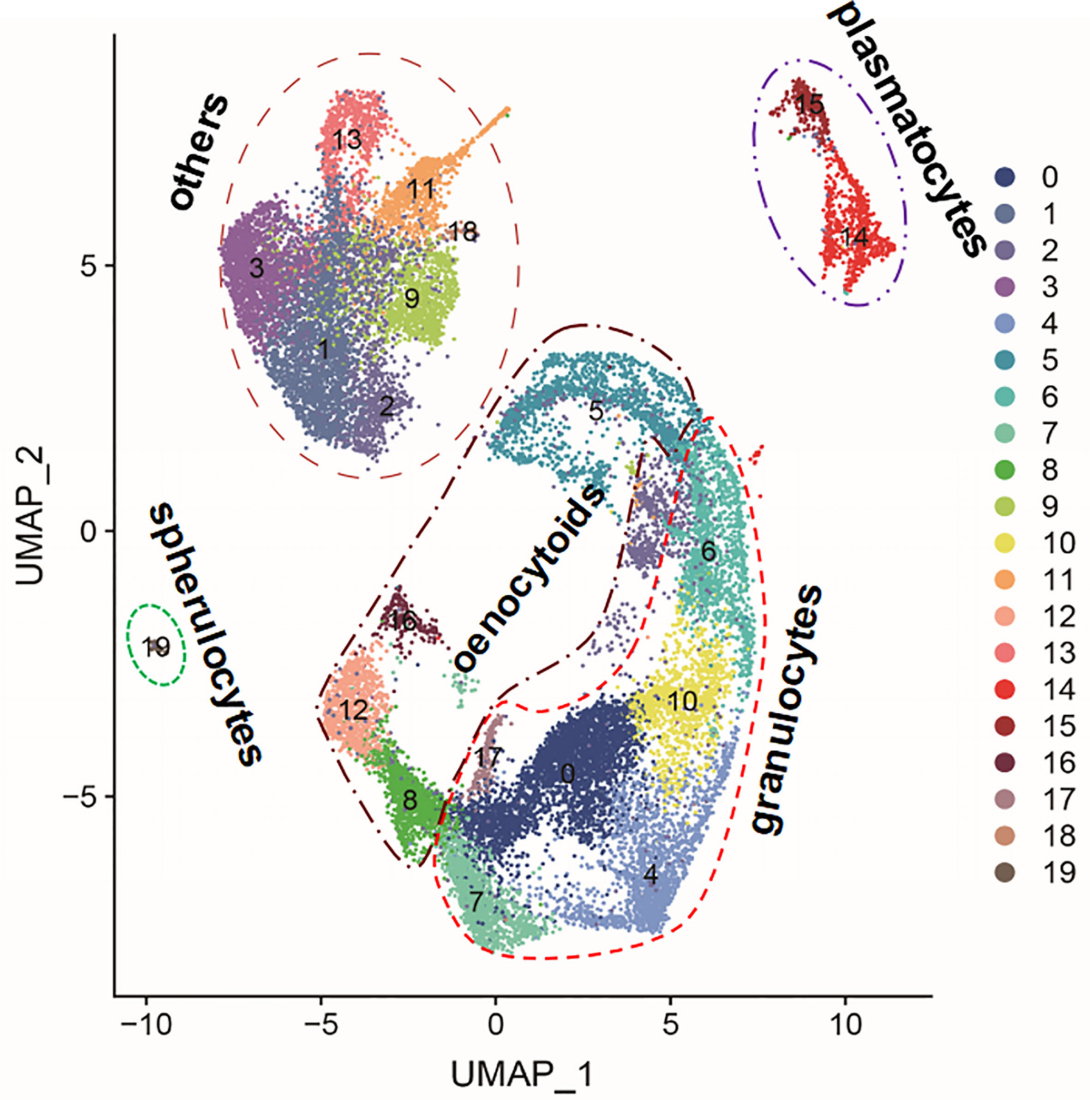
Uniform Manifold Approximation and Projection for Dimension Reduction (UMAP) plot of the 20 hemocyte clusters identified in silkworm larvae by scRNA-seq (24). Indicated is the grouping of the clusters in the four differentiated hemocyte cell types that were proposed in the literature (granulocytes: clusters 0, 4, 6, 7, 10 and 17; plasmatocytes: clusters 14 and 15; oenocytoids: clusters 5, 8, 12 and 16; and spherulocytes: cluster 19). Clusters that were heavily infected by baculovirus (>96%; **Table 1**) (others: clusters 1, 2, 3, 9, 11, 13 and 18) were not included in the analysis.

Although in the analysis of the scRNA-seq of silkworm hemocytes 20 clusters or subpopulations were identified, 7 of these clusters consisted of cells that were heavily infected by baculovirus (> 96%; **Table 1**; 24). An overview of the identified clusters (UMAP plot; 24) together with their proposed subtype identity (granulocyte, oenocytoid, plasmatocyte, spherulocyte) is presented in **Figure 1**. Pseudo-temporal ordering and morphological staining led to the proposal that the clusters in the BmNPV-infected group could correspond to pro-hemocytes that were released from the hematopoietic organs as a response to the depletion of differentiated hemocyte cell types by baculovirus infection (clusters referred to as “others” in **Figure 1**; 24). However, it can be assumed that baculovirus infection has a major impact on the physiology of the cells and that the transcriptome analysis may not provide a clear picture of the characteristics of pro-hemocytes in healthy silkworms. Another transcriptome analysis revealed large changes in infected hemocytes regarding amino-acid, carbohydrate, nucleic acid and lipid metabolism, conform to the expectation that infected hemocytes are transformed into virion production factories (35). Because of this limitation, the analysis is focused on the 13 clusters that encompass the differentiated cell types of the silkworm hemocyte population (granulocytes, oenocytoids, plasmatocytes and spherulocytes).

### 4.1 Cluster 0 (“Granulocyte”)

Cluster 0 represents the largest group of hemocytes that was separated based on gene expression levels. The levels of differential expression are relatively mild (log2FC < 2.7) which is indicative of an intermediary cell type. The selected marker genes of cluster 0 (**Table 2**) reflect the function of hemocytes in tissue remodeling, phagocytosis, regulation of oxidant stress and possibly melanization.

**Table 2:** Marker genes of cluster 0 (“granulocyte”).

Gene ID	Gene Name	Description	Log2FC	Indicated Process
BMSK0012279	UPB1	aliphatic nitrilase	2.73	tissue repair
BMSK0005620	–	5-hydroxytryptamine receptor	2.56	phagocytosis?
BMSK0009508	–	apolipoprotein D	2.33	(oxidant) stress response
BMSK0011099	FDPS	farnesyl diphosphate synthase	2.32	(oxidant) stress response?
BMSK0008561	GstZ1	glutathione transferase zeta	2.28	(oxidant) stress response
BMSK0007503	LITAF	lipopolysaccharide-induced tumor necrosis factor-alpha factor homolog	2.11	phagocytosis

Aliphatic nitrilase with β-ureidopropionase activity is involved in the catabolism of pyrimidine and the production of β-alanine (36) that has a role in cuticular tanning and sclerotization (37). The relatively high levels of 5-hydroxy-tryptamine (serotonin) receptor may reflect the regulation of phagocytosis by serotonin, as is observed in *Drosophila* (38, 39). Interestingly, another gene with high log2FC ranking is characterized with a LPS-induced TNF-activating factor (LITAF) domain that mediates targeting to intracellular membranes to regulate endosomal trafficking (40). In the malaria mosquito *Anopheles gambiae*, LITAF-like factors play a role in the regulation of phagocytosis (41) and are required for hemocyte differentiation into granulocytes and oenocytoids during invasion of the haemocoel by the malarial parasite (42).

Three other markers of this cluster may have a role in antioxidant signaling and stress protection. Such a role was already indicated for apolipoprotein D in the silkworm (43). Farnesyl diphosphate synthase is responsible for the synthesis of isoprenoids of which coenzyme Q may be of relevance because of its antioxidant activity and protection against reactive oxygen species (ROS) (44, 45). Finally, glutathione S-transferases (GST) of the zeta class are thought to represent an ancestral class of GST enzymes that play an important role in intermediary metabolism, i.e. the catabolism of tyrosine and phenylalanine (46, 47). Also this enzyme is thought to have a function in the protection against oxidant stress (48); moreover, by regulating tyrosine levels, it could play a role in the process of melanization (49).

All the above marker genes are also expressed in cluster 4, albeit with considerably lower ranking in log2FC values. This could indicate a developmental relationship between clusters 0 and 4. The gene encoding a LITAF-domain is also relatively highly differentially expressed in cells of cluster 15 (log2FC = 2.19).

### 4.2 Cluster 4 (“Granulocyte”)

Cells of cluster 4 contain more strongly upregulated genes (log2FC < 4.3) than several other clusters classified as granulocytes (e.g. clusters 0, 6 and 7) (**Table 3**). All the identified marker genes in cluster 4 are related to proliferation and mitosis, indicating their correspondence to pro-hemocytes or proliferating granulocytes.

**Table 3:** Marker genes of cluster 4 (“granulocyte”).

Gene ID	Gene Name	Description	Log2FC	Indicated Process
BMSK0001683	–	stathmin isoform X1	4.33	cell division
BMSK0005503	ncd	kinesin-like protein Ncd	3.55	cell division
BMSK0012933	KIF18A	kinesin-like protein KIF18A isoform X1	3.43	cell division
BMSK0012419	mad2l1-2,mad2l1-1	mitotic spindle assembly checkpoint protein MAD2A	3.38	cell division
BMSK0002666	–	uncharacterized protein LOC101740936	3.35	cell division
BMSK0014508	–	uncharacterized protein LOC101738390	3.26	cell division?

Stathmin is involved in the regulation of microtubule dynamics and acts as a microtubule destabilizing factor (50). As such it is proposed to be involved in the regulation of mitosis in the cell cycle and the cytoskeleton (51). Similarly, mitotic spindle assembly checkpoint protein MAD2A is a key component of the spindle assembly checkpoint during mitosis which is essential to maintain genomic stability during cell division (52). Ncd is a kinesin-like protein that is required for chromosome segregation in meiosis and in early mitotic embryonic divisions in *Drosophila* (53). Another kinesin-like protein, KIF18A, corresponds to a microtubule depolymerase with a role in chromosome congression to form the metaphase plate during mitosis (54). Furthermore, LOC101738390 is a homolog of the mitotic spindle and nuclear protein Mink in *Drosophila* (55). No clear function was revealed for uncharacterized protein LOC101740936, but HHPred analysis (56) revealed the existence of a short sequence (ENTPPHSH) that is also present in the linker sequence of a peptide that interacts with the catalytic and substrate recognition sites in the CDK2/cyclin A complex, a kinase that is active in the cell cycle S phase (57). However, no substrate recruitment site (RXL) or CDK2/cyclin A consensus phosphorylation site (S/TPKK) motifs were found in the vicinity and the presence of the sequence in uncharacterized protein LOC101738390 may be fortuitous.

During single cell RNAseq of *Drosophila* hemocytes, Ncd was identified as one of the marker genes of the PL-prolif cluster that are considered the mitotic precursor cells for most of the other hemocytes (4). Interestingly, both *mitotic spindle* and *nuclear protein* (*mink) and stathmin* (*stai*) were down-regulated in hemocytes following infection with Gram-positive bacteria in *Drosophila*, indicating that cell-cycle arrest was part of the response (58).

None of the marker genes in cluster 4 was differentially expressed in the remainder of the clusters, with the exception of cluster 14, where Ncd, KIF18A and uncharacterized protein 101740936 were marginally upregulated (log2FC value close to 1 in each case).

### 4.3 Cluster 5 (“Oenocytoid”)

Log2FC values of DEGs in cells of cluster 5 range from 0.36 to 3.37, indicating an intermediate level of specialization/differentiation (**Table 4**).

**Table 4:** Marker genes of cluster 5 (“oenocytoid”).

Gene ID	Gene Name	Description	Log2FC	Indicated Process
BMSK0015709	l(2)efl	heat shock protein hsp20.1	3.37	(oxidant) stress response
BMSK0001539	–	uncharacterized protein LOC101739676	3.36	tissue repair
BMSK0015595	l(2)efl	alpha-crystallin, partial	3.28	(oxidant) stress response
BMSK0015592	l(2)efl	heat shock protein hsp 19.9	3.21	(oxidant) stress response
BMSK0015594	l(2)efl	heat shock protein 20.8	3.16	(oxidant) stress response

The cells of cluster 5 are characterized by the significant induction of small heat shock protein (sHSP) genes (4 out of 5 top markers with log2FC from 3.16 to 3.37). Interestingly, the sHSP genes belong to two closely located clusters on chromosome 27, in which other sHSPs (hsp23.7; log2FC = 1.31) and HSPs (HSP68, log2FC = 2.52; HSP83, log2FC = 2.39) are also up-regulated. Additionally, expression of two other sHSP genes, hsp12.2 and α-crystallin B, that are located on chromosome 5, are increased (log2FC = 2.34 and 2.21, respectively).

The family of sHSPs is characterized by a conserved domain of about 100 residues, the α-crystallin domain, at their C-termini (59). sHSPs are abundant in conditions of stress and act as chaperone proteins. Increased production of sHSPs in hemocytes is linked to the protection against oxidative stress, e.g. after wounding or infection (60, 61). The protective function of HSPs relates to the reduction of damage from reactive oxygen species (ROS) that are produced in response to wounding and that play a role in melanization and clotting (62).

The association with ROS production is consistent with the classification of cells of cluster 5 as oenocytoids (24). Consistent with this proposition, increased expression of both prophenoloxidase genes, involved in melanization, is indeed observed (log2FC of 2.44 and 2.76).

The sequence of uncharacterized protein LOC101739676 (118 AA) corresponds to a LU domain or Ly6 module, that consists of approximately 80 amino acids and contains a conserved pattern of cysteine residues forming disulfide bridges to create the three-fingered structural motif (63, 64). The Ly6 module is found in both soluble and membrane-anchored proteins and functions as a protein-protein interaction domain that has been adopted in many different biological processes. Interestingly, a Ly6 module-containing membrane-anchored protein, Retroactive, is required for the organization of chitin fibers at the epidermal surface during cuticle assembly in larvae of *Drosophila* (65). Another Ly6 protein, Coiled, contributes to septate junction assembly in epithelial tissues as well as in glial cells to insulate the central nervous system from the hemolymph (66, 67). In mammals, Ly6 proteins are also known to modulate nicotinic acetylcholine receptors, voltage-gated potassium channels and Wnt/β-catenin signaling (68–70). The presence of Ly6 proteins in hemocytes is considered consistent with a role in tissue (wound) repair, encapsulation of pathogens and immune signaling.

All the marker genes of cluster 5, as well as HSP68, HSP83 and sHSP12.2 on chromosome 5, are also differentially expressed in cells of cluster 16, which are also tagged as oenocytoids (24), albeit with lower log2FC (range: 1.94 - 2.63) and lower DEG ranking (range: 34 -102). However, α-crystallin B is proposed as the top marker gene of cluster 16 (log2FC = 5.50; see further below). Two out of five marker genes of cluster 5 are also increased in clusters 6, 8 and 12 (the latter two also marked as oenocytoids; 24) but with low log2FC (range: 0.79 – 1.51). In addition, sHSP20.1 on chromosome 27 and sHSP12.2 on chromosome 5 have high log2FC values in cluster 19 (6.08 and 5.10, respectively; corresponding to relatively low respective DEG ranking of 45 and 42).

### 4.4 Cluster 6 (“Granulocyte”)

Also for cluster 6, log2FC values remained moderate and the cluster is therefore proposed to represent a precursor or intermediate cell type (**Table 5**).

**Table 5:** Marker genes of cluster 6 (“granulocyte”).

Gene ID	Gene Name	Description	Log2FC	Indicated Process
BMSK0001430	E(spl)mbeta-HLH	enhancer of split mbeta, partial	2.98	proliferation and growth
BMSK0006701	Myc	c-myc isoform X1	2.48	proliferation and growth
BMSK0006208	SNU13	cleavage and polyadenylation specific factor 4	2.39	proliferation and growth
BMSK0014249	NIP7	60S ribosome subunit biogenesis protein NIP7 homolog	2.39	proliferation and growth
BMSK0000891	–	nucleolin	2.38	proliferation and growth
BMSK0012073	FKBP45	FK506-binding protein 45	2.27	proliferation and growth?

Indeed, all identified marker genes of cluster 6 seem to be related to proliferation and growth. Most conspicuously, the second marker gene encodes Myc, a basic helix-loop-helix (bHLH) transcription factor that regulates the expression of a large number of genes mainly involved in growth and cell proliferation (71). Myc is involved in the regulation of the growth and size of the hematopoietic lymph gland in *Drosophila* (72) and the Myc pathway is enriched in a subset of pro-hemocytes with stem cell-like properties (PH1) that reside in the lymph gland (23).

Furthermore, the gene encoding another bHLH transcription factor, Enhancer of Split (E(spl), mbeta isoform), had the highest level of differential expression (log2FC = 3.0). bHLH proteins encoded by the E(spl) complex are target genes of Notch signaling that guide cell specification (73). Interestingly, the stem-like pro-hemocytes (PH1 subtype) in *Drosophila* mentioned above also preferentially express Notch, its ligand Delta, and the E(spl) target genes (23), reinforcing the idea of stem cell-like properties of cluster 6 hemocytes in *Bombyx*.

Notch signaling also controls crystal cell differentiation (74, 75) and the Notch target gene *E(spl)m3-HLH* is upregulated in immature cells of the crystal cell lineage (5). It can therefore be proposed that cluster 6 cells may represent the precursor lineage of the oenocytoid-type hemocytes in *Bombyx*, which are thought to have similar functions as crystal cells in *Drosophila* (1). However, the characteristic prophenoloxidase marker gene of crystal cells/oenocytoids is not expressed in cluster 6 cells, conform to the immature or intermediate state of the cells. Although cluster 6 hemocytes originally were categorized as granulocytes (24), more validation studies are needed with respect to the differentiation status of this hemocyte cell type.

Other genes that are highly differentially expressed can also be associated with growth and proliferation. Cleavage and polyadenylation specific factor 4 (CPSF4, SNU13) is involved in mRNA 3’-end processing and polyadenylation (76). CPSF4 plays a role in cancer cell survival and proliferation through modulation of signaling pathways (77) and regulation of gene expression (78). Interestingly, depletion of a factor of the CPSF complex could suppress the growth of the lymph gland in *Drosophila*, possibly through aberrant 3’-end processing of histone mRNAs (79).

Nucleolin is known as one of the most abundant (non-ribosomal) proteins in the nucleolus but can have many other functions, most notably as a histone chaperone to regulate chromatin remodeling and the facilitation of the passage of RNA polymerase II through nucleosome complexes (80, 81). The importance of nucleolar function is underlined by the enrichment of nucleolar pre-rRNA processing protein NIP7 that is involved in ribosomal large subunit biogenesis.

Another protein with high log2FC is FK506-binding protein 45 (FKBP45), which belongs to the immunophilin family of proteins that have peptidylprolyl isomerase activity and function as protein chaperones (82). In *Spodoptera* Sf9 cells, FKBP46 is the substrate of Sf caspase-1 during activation of apoptosis, which is inhibited by baculovirus-derived p35 protein during infection (83). While homology between *Bombyx* FKBP45 and *Drosophila* FKBP39 (closest homolog) seems to be limited to the FK506-binding domain, it was noted that FKBP39 has a central highly charged region (also present in FKBP45) that allows its quarternary organization in large multifunctional complexes (with unknown function) in the nucleolus, at nucleosomes and at microtubules (82). Whether such plasticity and flexibility in function also applies to *Bombyx* FKBP45, remains to be determined.

Cluster 5, 10 and 14 each have 5 marker genes of cluster 6 as DEGs but generally with much lower log2FC (range: 0.56 – 1.61). Myc, NIP7, nucleolin and FKBP46 are common as DEGs among clusters 5, 10 and 14. SNU13 as DEG is also observed clusters 10 and 14. E(spl) has a relatively high log2FC of 2.35 in cluster 5.

### 4.5 Cluster 7 (“Granulocyte”)

Also for the cells of cluster 7, DEG values are moderate (log2FC < 3.1), indicating an intermediate or possibly regulatory cell type (**Table 6**). Several identified marker genes (cathepsin B, metalloproteinase inhibitor, carboxylesterase) indicate a function in wound healing or tissue remodeling.

**Table 6:** Marker genes of cluster 7 (“granulocyte”).

Gene ID	Gene Name	Description	Log2FC	Indicated Process
BMSK0010120	Ctsb	cathepsin B precursor	3.08	tissue repair
BMSK0001921	–	uncharacterized protein LOC101739791	2.88	mitochondrial function respiration
BMSK0011976	SLC39A3	zinc transporter ZIP3	2.85	tissue repair
BMSK0012983	–	inducible metalloproteinase inhibitor protein-like	2.82	tissue repair
BMSK0003260	NLGN1	carboxylesterase clade H	2.76	tissue repair

The top marker, cathepsin B, is a member of lysosomal cysteine proteases of the papain superfamily that have the unique capability to act as both endo- and exopeptidases. However, cathepsin B can also be found outside of the lysosomal compartment and be located in the cytosol, at the plasma membrane and in the pericellular environment (84). In mammals, a regulatory function in innate immunity is implicated for cathepsin B because of its role in the processing and trafficking of cytokines such as interleukin-1β and tumor necrosis factor α (85). In the flesh fly, *Sarcophaga peregrina*, cathepsin B is secreted by pupal hemocytes and causes the dissociation of the fat body during metamorphosis (86). Also in *B. mori*, expression of cathepsin B in hemocytes was proposed to play a role in the regulation of metamorphosis (87). Thus, cathepsin B produced by cluster 7 is predicted to have a role in tissue remodeling or the innate immune response.

The second marker (“uncharacterized protein LOC101739791”) corresponds to a homolog of the mitochondrial complex I intermediate-associated protein 30 (CIA30) that has been shown in *Drosophila* to be a chaperone for the assembly of mitochondrial complex I (88). Mitochondrial complex I (NADH:ubiquinone oxidoreductase/NADH dehydrogenase) is also involved in the regulation of oxidative stress and is considered as a main site for ROS production (89) that play a role as signaling molecules to activate various pathways including mitogen-activated protein kinase (MAPK) and phosphoinositide 3-kinase (PI3K) (90, 91). ROS signaling has been implicated in several homeostatic processes such as wound healing, cell differentiation and innate immunity (92). During immune defense, ROS can be released as effectors by macrophages to damage cellular structures of invading pathogens (93). The increased expression of an assembly factor for mitochondrial complex I may therefore reflect the increased importance of ROS production by mitochondria in cluster 7 hemocytes in the silkworm.

Two proposed markers are related to the function of metalloproteinases: an inducible physiological metalloproteinase inhibitor and the zinc transporter ZIP3. Metalloproteinases are well known for degrading extracellular matrix proteins (collagens, proteoglycans, laminins) but can also cleave a variety of other extracellular substrates and therefore exhibit pleiotropic roles as regulators of extracellular signaling (94, 95). In *Drosophila*, matrix metalloproteinases are essential for tissue remodeling during metamorphosis (96). The increased expression of inhibitor and transporter genes in cluster 7 hemocytes suggests the presence of mechanisms to regulate metalloproteinase activity either through direct interaction or controlling the availability of zinc ions, that are essential for the catalytic mechanism (97). Zinc transport has been found to be essential for remodeling of fat body tissue by matrix metalloproteases in *Drosophila* (98). In Lepidoptera, it was demonstrated that hemocyte behavior and function was affected by the presence of zinc in the culture medium, implicating it as a regulator of metalloprotease function (99).

Carboxylesterases form a large superfamily and have important roles in xenobiotic metabolism (including insecticide resistance), pheromone inactivation and regulation of development and neurogenesis (100). The proposed carboxylesterase marker of cluster 7 hemocytes belongs to clade H that is associated with the secreted catalytic class and has single orthologs among the silkworm, *Drosophila*, the honeybee, the malaria mosquito and the Colorado potato beetle (101, 102). No clear function has been assigned to clade H carboxylesterases. Microarray data show preferential expression in hemocytes and head tissue in *Bombyx* (101); expression of the *Drosophila* homolog (CG5397) in larval plasmatocytes was also reported (103). Because of its relatedness with the *Drosophila* glutactin clade, clade H carboxylesterases may play a role in cell segregation and adhesion.

Two marker genes of cluster 7 (ZIP3, carboxylesterase H) are also differentially expressed in clusters 0, 4 and 10; in addition, cathepsin B expression is also enriched in clusters 0 and 4. Levels of differential expression of the three genes decline gradually from clusters 7 (highest), 0, 4 and 10 (lowest). The data reinforce the developmental relationship between clusters 0 and 4.

### 4.6 Cluster 8 (“Oenocytoid”)

Also the cells of cluster 8 do not seem to be highly differentiated as evidenced by the relatively low log2FC values of the DEGs (range: 0.36 to 2.30) (**Table 7**).

**Table 7:** Marker genes of cluster 8 (“oenocytoid”).

Gene ID	Gene Name	Description	Log2FC	Indicated Process
BMSK0014159	21G1	30K protein 21	2.30	pattern recognition
BMSK0013762	21G1	30K protein 16	2.30	pattern recognition
BMSK0003871	hyi	hydroxypyruvate isomerase	2.20	carbohydrate metabolism
BMSK0014158	19G1	microvitellogenin	2.18	pattern recognition
BMSK0012226	–	uncharacterized protein LOC101743414	2.17	gene expression

Three of the five marker genes encode 30K proteins (genes indicated as 19G1 and 21G1) and a fourth 30K protein gene (21G1, 30K protein 19) has also a high log2FC of 2.14. 30K proteins were initially identified as major proteins in the hemolymph that are synthesized by the fat body during the larval stage and become absorbed by the oocyte as yolk proteins in the pupal stage (104, 105). Altogether forty-six 30K proteins were identified in *Bombyx mori* that were classified as low molecular weight lipoproteins (lipoprotein 11 family), although their lipid content remains unknown (106). The four genes that are differentially expressed in cluster 8 hemocytes all encode “typical” 30K proteins (belonging to clusters III and IV; 106, 107). Interestingly, the four genes belong to two close loci on chromosome 24.

However, besides their role as nutrient proteins, 30K proteins have also been shown to be involved in inhibition of apoptosis (108) and immune defense (109). More specifically, 30K proteins can bind to fungal glucans and therefore function as pattern-recognition receptors (6G1, 19G1 and 21G1 genes; 110, 111). In addition, specific 30K proteins become induced after injection of fungal wall glucans and can promote encapsulation by hemocytes (109).

Hydroxypyruvate isomerase (HYI) catalyzes the conversion of hydroxypyruvate to 2-hydroxy-3-oxopropanoate (also known as tartronate semi-aldehyde) and therefore may be involved in carbohydrate transport and metabolism (112). HYI activity links with the glyoxylate cycle and serine metabolism. Expression of HYI was also observed in crystal cells of *Drosophila* (103).

Finally, HHpred analysis (56) reveals that uncharacterized protein LOC101743414 contains a short Glutamine-rich region of 87 amino-acids that is similar to subunit TRAP230 of the thyroid hormone receptor-associated protein complex. The TRAP complex mediates the interaction between specific transcriptional activators and the general transcription machinery (113). The available evidence therefore indicates that uncharacterized LOC101743414 has a role in the regulation of transcriptional activation.

Conform to its classification as oenocytoids (24), cluster 8 also displays induction of the two prophenoloxidase genes (log2FCs 2.17 and 1.75; DEG rankings 3 and 10).

HYI and the four 30K genes are up-regulated in all clusters that were identified as oenocytoids (clusters 5, 8, 12 and 16). While log2FCs in cluster 5 are below 2, higher values (range: 2.36 to 3.10) are observed in clusters 12 and 16 but these are associated with a much lower DEG ranking than in cluster 8 (range 13 to 26 for cluster 12; range 38 to 78 for cluster 16). By contrast, LOC101743414 is not a DEG in the other oenocytoid-like cells although it is up-regulated in a few other clusters that are not classified as oenocytoids [clusters 0, 4, 7 and 17 with low to moderate log2FC (0.73 to 2.38)].

With low log2FC < 1, HYI and the four 30K genes are also DEGs in cluster 7. It is also noted that another 30K gene (BMSK0011510), located on chromosome 20, is expressed in hemocytes of clusters 0, 7, 10, 14 and 15 but at DEG ranking > 100.

### 4.7 Cluster 10 (“Granulocyte”)

The 5 selected marker genes of cluster 10 have relatively high levels of differential expression (log2FC values between 2.7 and 4.7) (**Table 8**) while the other DEGs have moderate to low values (such as clusters 0, 6 and 7).

**Table 8:** Marker genes of cluster 10 (“granulocyte”).

Gene ID	Gene Name	Description	Log2FC	Indicated Process
BMSK0013794	IMP-L2	insulin-related peptide binding protein	4.70	metabolism
BMSK0001262	–	uncharacterized protein LOC106126609	4.17	regulation of biquitination?
BMSK0015401	CECB1,CECB2	Cecropin family	3.04	antimicrobial peptide tissue repair?
BMSK0005248	–	–	2.76	regulation of cell shape?
BMSK0005114	alpha-Man-Ia	mannosyl-oligosaccharide alpha-1,2-mannosidase isoform A isoform X2	2.68	protein glycosylation

The highest ranked gene encodes the homolog of Imaginal Morphogenesis Protein-Late 2 (IMP-L2), a secreted factor and member of the immunoglobulin family (114). IMP-L2 binds insulin-like peptides and functions as an antagonist of insulin/insulin-like growth factor signaling (115). As such, IMP-L2 regulates developmental timing and could be involved in the regulation of aerobic glycolysis in hemocytes during the immune response (116).

Cluster 10 cells are also characterized by the increased expression of another secreted protein, the antimicrobial peptide Cecropin B (117). Actually, 6 different Cecropin genes belonging to the same cluster on chromosome 26 and with identical amino-acid sequence are upregulated in cluster 10 (with log2FC of 3.0, 2.6, 2.4, 2.1, 2.1, and 1.9). While the function of Cecropin as an inducible lytic peptide against bacterial membranes is well established (118), it is noted that Cecropins can also have a regulatory function in developmental processes such as cuticle formation *via* the regulation of prophenoloxidase expression (119).

The increased expression of IMP-L2 and Cecropins indicates that cluster 10 may be associated with humoral immunity or have a regulatory function by the secretion of cytokines. To support the latter, it is noted that paralytic peptide, a cytokine with diverse functions in growth and immunity (120), is also upregulated in cluster 9 (log2FC = 2.28). Another marker gene in cluster 10 encodes mannosyl-oligosaccharide alpha-1,2-mannosidase IA that participates in the maturation process of N-glycans during protein glycosylation in the Golgi complex (121). Protein glycosylation is most often associated with secreted and membrane proteins. Interestingly, expression of alpha-1,2-mannosidase I variants was associated with stress resistance in *Drosophila* (122).

The two other remaining marker genes correspond to unknown proteins. A region in LOC106126609 (E value 0.9 in HHpred analysis) resembles the UBA domain that is found in ubiquitin-binding proteins. The other sequence is likely incomplete and resembles a region in proteins of the major intrinsic protein (MIP) family that function as membrane channels that selectively transport water, small neutral molecules, and ions. Aquaporins belong to the MIP family and are involved in the regulation of cell shape during cellular immunity in *Spodoptera exigua* (123). The aquaporin Prip is one of the most enriched genes in plasmatocytes of *Drosophila* (58) and another aquaporin, Drip, is enriched in lamellocytes (5).

Four cecropin genes from the same cluster are also moderately upregulated in cluster 4 (log2FC between 1.7 and 2.0). Increase in expression for two cecropins of this cluster is also found in cluster 17 (log2FC of 1.1 and 2.1). One of the cecropins in this cluster (BMSK0015395) is up-regulated in clusters 0, 4, 7, 10 and 17 (log2FC of 1.32, 1.90, 1.76, 1.87 and 1.15, respectively). On the other hand, high induction of one cecropin (Cecropin A) is observed in cluster 15 (log2FC = 5.4 while ranked 30 as DEG) but this gene is located in another location in the genome on chromosome 6.

### 4.8 Cluster 12 (“Oenocytoid”)

Thirteen DEGs in cluster 12 have a log2FC value of >4, which could be indicative of higher differentiation levels. (**Table 9**).

**Table 9:** Marker genes of cluster 12 (“oenocytoid”).

Gene ID	Gene Name	Description	Log2FC	Indicated Process
BMSK0002107	MKX	homeobox protein Mohawk isoform X1	6.35	tissue repair?
BMSK0002756	CFAP57	cilia- and flagella-associated protein 57 isoform X2	6.14	tissue repair?
BMSK0013258	IAP	inhibitor of apoptosis protein isoform X1	4.77	regulator of apoptosis
BMSK0009087	–	uncharacterized protein LOC114246272	4.74	dimerization domain?
BMSK0013854	Trx-2	ABJ97191.1 thioredoxin-like protein	4.74	(antioxidant) stress response
BMSK0014801	–	trichohyalin-like	3.30	regulator of cell shape

*Mohawk* is a member of the TALE class of atypical homeobox genes that functions as a potent transcriptional repressor (124). In mammals, Mohawk is a tendon-specific transcription factor that regulates collagen expression (125). It can be suggested that Mohawk may be a regulator of hemocyte-specific functions in tissue remodeling (126).

Cilia- and flagella-associated protein 57 (CFAP57) belongs to the WD repeat-containing proteins that function in the assembly of large protein complexes (127). More specifically, CFAP57 is localized in the axoneme of cilia in human nasal epithelial cells and mutations in the green alga *Chlamydomonas* cause ciliary dyskinesia (128). Interestingly, an involvement of CFAP57 (previously known as WDR65) in tissue remodeling (craniofacial development) was also implicated (129). Thus, CFAP57 is predicted to be involved in changes in hemocyte morphology, such as elongations, that are reminiscent of the function of lamellocytes to engulf large foreign bodies such as parasitoid eggs in *Drosophila* (130). Interestingly, in larvae of particular drosophilids, a specialized hemocyte type, the nematocyte, can be observed that have spindle-like projections with high densities of mitochondria and microtubules (131), which may be considered as cilia-like structures. However, such extreme cell types have not been described among hemocytes in *Bombyx mori* so far.

Also trichohyalin is predicted to have a primary role in cytoskeleton dynamics. Trichohyalin is a keratin-binding protein that mediates its assembly to keratin filaments in mammals (132). While insects lack keratins and intermediary filaments are also thought to be absent (133), it is nevertheless tempting to speculate a role for trichohyalin in tissue remodeling and the encapsulation reaction during immune defense, perhaps by interacting with other skeletal elements such as cuticular building blocks. Genes encoding cuticular proteins are indeed induced in several other clusters (but not in cluster 7), the highest in cluster 15 (log2FC = 6.75).

IAP (inhibitor of apoptosis protein) is a critical regulator of cell survival by its capacity to inhibit apoptosis (134). However, IAP proteins are also involved in the regulation of innate immune signaling pathways (135), e.g. the Imd pathway that is activated by Gram-negative bacteria (136). Increased IAP expression may reflect the activation of innate immunity and the promotion of a state of inflammation.

The fourth marker gene encodes an uncharacterized protein of 239 amino-acids (LOC114246272). HHpred analysis (56) reveals a region with a seven residue sequence repeat found in coiled coil regions that are involved in protein dimerization (137). The final marker, on the other hand, corresponds to Thioredoxin-2 (Trx-2), a small heat-stable protein that contains a redox-active disulfide with anti-oxidative function (138). Trx-2 becomes induced by various types of stress and, interestingly, provides resistance against nucleopolyhedrovirus infection in *Helicoverpa armigera* (53).

It is also noted that prophenoloxidase 1 and 2 are among the 25 highest ranking DEGs (log2FC of 2.65 and 2.07, respectively) in the cells of cluster 12, which therefore may also be involved in the melanization reactions that regularly follow the encapsulation of macro-pathogens (139), consistent with the classification as oenocytoids (24). Interestingly, in *Drosophila*, the Runt-related transcription factor Lozenge is a marker of crystal cells (140) and, similarly, Runt-related transcription factor 3 is also significantly induced (log2FC = 2.65) in cluster 12, possibly indicating conservation of hemocyte differentiation programs between *Drosophila* and *Bombyx*. However, in contrast to our analysis, the Runt-related transcription factor Lozenge was also found to be highly expressed in hemocytes of the silkworm and its role in the melanization reaction by hemocytes was indicated by over-expression and gene silencing experiments (141).

Another interesting observation is the significant upregulation of the nuclear hormone receptor HR3 (log2FC = 4.51 and 3.25 for 2 isoforms), which is a part of the regulatory cascade initiated by 20-hydroxy-ecdysone (20E) (142). 20E controls the Imd innate immune pathway in *Drosophila*; more specifically, RNAi of HR3 decreases the induction of antibacterial peptide (AMP) genes by peptidoglycan (143). In hemocytes of larvae of *Heliothis virescens*, HR3 becomes induced during baculovirus infection (144). Similar to the increased levels of IAP, stimulation by 20E signaling in the cells of cluster 12 may be related to the activation of the innate immune response.

All the marker genes, with the exception of CFAP57, are also increased in the cells of cluster 16, but with much lower log2FC (range: 0.47 - 3.04). In addition, the two prophenoloxidase genes are significantly induced in clusters 5, 8 and 16 (log2FC range: 1.75 to 3.04), consistent with the proposition that clusters 5, 8, 12 and 16 correspond to oenocytoids (24). The two prophenoloxidases also appear as DEGs in cluster 7 but with much lower log2FC (0.69 and 0.64). On the other hand, Runt-related transcription factor 3 is differentially expressed in clusters 5, 12 and 16 (log2FC values of 2.43, 2.65 and 1.21, respectively), but not in cluster 8. Runt-related transcription factor 3 also appears ad DEG in cluster 6 (log2FC = 1.79).

### 4.9 Cluster 14 (“Plasmatocyte”)

DEGs in cluster 14 have high log2FC values (7 genes with log 2FC > 5; 18 genes with 5 > log2FC > 4; 26 genes with 4 > log2FC > 3), which indicates a high level of differentiation (**Table 10**).

**Table 10:** Marker genes of cluster 14 (“plasmatocyte”).

Gene ID	Gene Name	Description	Log2FC	Indicated Process
BMSK0013803	–	ommochrome-binding protein	9.72	(antioxidant) stress response
BMSK0012809	–	antitrypsin isoform 3	6.30	regulation of melanization
BMSK0010812	–	insulin-like peptide receptor	5.40	decoy receptor for insulin
BMSK0005347	Itga1	integrin alpha-IIb-like precursor	5.37	aggregation/encapsulation
BMSK0013701	–	uncharacterized protein LOC101747033	5.35	encapsulation?

A gene encoding an ommochrome-binding protein is differentially expressed at high levels (log2FC = 9.72) in the cells of cluster 14. In insects, ommochrome synthesis represents the most important pathway for the removal of tryptophan metabolites, that are toxic when present in high amounts (145). In addition, ommochromes also serve as eye and integument pigments (146, 147). Secreted ommochrome-binding proteins therefore may function as ommochrome carriers in the hemolymph to sites of excretion (hindgut, Malpighian tubules) or storage in pigment granules (eye, integument). Ommochrome-binding proteins are also synthesized by the fat body and accumulate in the hemolymph at their highest levels by the end of larval life (148). On the other hand, because of the capacity of ommochromes to buffer oxidative stress (149), ommochrome-binding proteins may also be linked to oxidative metabolism and protect hemocytes from excessive production of ROS during the immune response.

The second DEG with high log2FC corresponds to antitrypsin, which belongs to the serpin family of protease inhibitors (150). The majority of serpins in insects has a role in innate immunity in which they function as negative regulators (151). Antitrypsin in *Bombyx* is the homolog of Serpin-1 in *Manduca sexta*, which has been the subject of several functional studies (150, 152). Serpin-1 isoforms were shown to block the proteinase cascades leading to the activation of the ligand of the Toll receptor, Spätzle (153), as well as the prophenoloxidase enzymes involved in melanization (154, 155).

A gene with the annotation “insulin-like peptide receptor” (BMSK0010812) is also proposed as a marker gene for cluster 14 and is highly induced. However, this gene does not correspond to the canonical “insulin receptor” (InR) gene of *Bombyx mori* (156; Gene ID = BMSK0002922). The amino-acid sequence is highly similar to the extracellular domain of insulin-like receptors and contains fibronectin type III and L domains (157–159). HHpred analysis (56) confirms the formation of a binding cavity that can interact with insulin-like peptides. Although no signal peptide is present, a transmembrane segment could be identified. In the absence of a tyrosine kinase domain, the “insulin-like peptide receptor” therefore represents a decoy receptor that binds insulin-like ligands efficiently but is not capable to activate a signaling pathway.

While the expression of integrin β3 in cluster 14 as a marker gene for plasmatocytes was already noted (log2FC=4.10; 24), its partner for heterodimerization, integrin αIIb is also considered as characteristic for this hemocyte type (log2FC=5.37). Interestingly, mammalian integrin αIIb has a specific function in platelets, where it joins with integrin β3 to generate a fibrinogen receptor (160). The integrin αIIb/β3 dimer is required for platelet aggregation in mammals (161) and can therefore be predicted to have a similar function in lepidopteran plasmatocytes/*Drosophila* lamellocytes for the encapsulation of foreign bodies and pathogens.

In *Manduca sexta*, specific antibodies or RNAi that inhibit integrin β1 result in the suppression of encapsulation by plasmatocytes (162). Similarly, integrin β1 plays a role in spreading and encapsulation by plasmatocytes in *Ostrinia furnacalis* (163, 164). In the silkworm, 11 integrin family members (6 α- and 5 β-subunits) were identified, of which the majority was preferentially expressed in the hemocytes (165). Looking at the differential expression of integrins among hemocyte clusters, it appears that integrins α3 and β1 have the widest expression although at relatively modest levels (log2FC ranges from 0.55 to 2.07). Integrin α3 can be detected as DEG in clusters 0, 4, 7, 8 and 15 while integrin β1 occurs in clusters 3, 7, 9, 11 and 15. In clusters 14 and 15, however, other integrins are expressed at high levels. As already mentioned, both αIIb and β3 integrins are uniquely and highly induced in cluster 14. Also in cluster 15, integrin αIIb (but not β3) is differentially expressed (log2FC=4.63); in addition, integrins α-PS4 and α-PS1 are induced (log2FC of 5.55 and 4.14, respectively. The high expression of unique integrins in clusters 14 and 15 may be related to their function as plasmatocytes (24). Interestingly, a recent study indicated a regulatory role for integrin β3 in the immune response because of its inhibitory effect on melanization and the expression of immune-related genes (166).

The last marker gene, encoding uncharacterized protein LOC101747033, corresponds to a fatty acyl-coA reductase (FAR) that catalyzes the synthesis fatty acyl alcohols (167). Besides a role in pheromone biosynthesis (168), FARs are engaged in the production of wax substances for the isolation of surfaces such as the inner lining of the tracheal tubes (169). The expression of FAR in plasmatocyte-like cells may be related to the function of encapsulation.

Cluster 14 cells also produce several secreted proteins such as promoting protein, that regulates the lipid composition and fluidity of the plasma-membrane (log2FC = 4.99; 170), and multifunctional peptides designated as “cytokines” that also regulate spreading behavior (growth-blocking peptide, log2FC = 4.67 and paralytic peptide, log2FC = 2.74; 171).

All proposed marker genes, with the exception of ommochrome-binding protein, are also highly induced in cluster 15 (log2FC range from 3.61 to 4.58) but with much lower DEG ranking (53 to 88). Promoting protein, growth-blocking peptide and paralytic peptide are also DEGs in cluster 15 (log2FC range from 2.28 to 4.87; DGE rank from 48 to 137). The overlap in DEGs confirms the close relation between clusters 14 and 15 as plasmatocytes (24). Regarding other clusters, paralytic peptide and promoting protein are also DEGs in cluster 10 (respective log2FC and DGE rank of 2.28 and 1.22, and 10 and 56).

### 4.10 Cluster 15 (“Plasmatocyte”)

Cluster 15 also represents a highly differentiated type of hemocytes with high levels of log2FC in DEGs. Forty-three and 46 DEGs have log2FC > 5 and 5 > log2FC > 3, respectively (**Table 11**).

**Table 11:** Marker genes of cluster 15 (“plasmatocyte”).

Gene ID	Gene Name	Description	Log2FC	Indicated Process
BMSK0010425	–	lectin 5 precursor	12.39	pattern recognition
BMSK0012020	Klkb1	serine protease snake-like	8.41	encapsulation
BMSK0013971	PPAF2	clip domain serine protease 11 precursor	7.64	melanization
BMSK0006713	–	immune-related protein	7.27	encapsulation?
BMSK0015665	–	KWMTBOMO16085	7.21	encapsulation?

The top DEG (log2FC > 12) in this cluster has a C-type lectin-like domain” (CTLD). C-type lectins were originally defined by their dependence on Ca^2+^ as well as by the presence of a so-called “carbohydrate-recognition domain” (CRD) but later studies indicated that such domains also occurred in many other proteins that do not bind calcium or carbohydrates (172). The designation as CTLD was subsequently introduced to solve the conundrum that CTLD proteins can bind a wide variety of ligands, including sugars, proteins, lipids and inorganic compounds (173).

The identified sequence with CTLD is small (93 amino-acids) and may be incomplete; a highly similar sequence from *B. mandarina* is larger (173 amino-acids) and has a signal peptide but no transmembrane domain. In *B. mori*, 23 genes with CTLD domains were identified (174). Several CTLD proteins from Lepidoptera can bind pathogen-associated molecular patterns in Gram-positive and -negative bacteria and fungi and are involved in the activation of the cellular immune response (175–179). Interestingly, the identified CTLD has significant homology (23% identity, 42% similarity) with mammalian DC-SIGN (CD209) that interacts with the envelope proteins of Dengue virus (180). Importantly, Dengue virus infection can be inhibited by soluble DC-SIGN proteins (181, 182). Taken into account that a significant proportion of cluster 15 cells are infected with baculovirus, it can be speculated that the increased expression of soluble CTLD may be involved in the binding of baculoviral envelope proteins as an antiviral defense mechanism. Of interest is the observation that plasmatocytes are more resistant than other hemocyte cell types against BmNPV infection in *Bombyx* (183).

Two other highly induced marker genes belong to the trypsin family of serine proteases (184). In the hemolymph, serine proteases are produced as zymogens that are successively activated in a cascade sequence, e.g. during clotting/coagulation and encapsulation/melanization. While factors regulating melanization have been identified earlier (185), less is known about the regulation of coagulation in insects (186). Genes belonging to both cascades are upregulated in cluster 15: serine protease snake-like is a homolog of kallikrein that is involved in clotting/coagulation while serine protease 11 corresponds to phenoloxidase-activating factor 2. In the silkworm hemolymph, both pathways become integrated in large multifunctional immune complexes following injury or pathogen infection (187). Besides the proposed marker genes, several other serine protease precursors are highly ranked DEGs: serine protease gd-like (log2FC = 7.20) and serine protease gd (log2FC = 5.20), associated with coagulation; and prophenoloxidase activating enzyme precursor (log2FC = 6.42), clip domain serine protease 11 precursor (log2FC = 6.31) and serine protease 7 (log2FC = 5.14), involved with melanization. Remarkably, β-1,3-glucan recognition protein, one of the triggers of the melanization cascade (188), is also induced (log2FC = 5.18).

The remaining marker genes encode secreted proteins (presence of signal peptide) with functions related to the extracellular matrix. The sequence of the immune-related protein (BMSK0006713) corresponds to a reeler or reelin motif (approximately 170 amino-acids) that occurs in extracellular matrix proteins that guide neuronal migration (189). KWMTBOMO16085, on the other hand, shows a short sequence that also occurs in the elastomeric protein glutenin (190). High expression of both proteins is consistent with a function in invagination or encapsulation that needs be more defined by further experimentation.

Following serine proteases are also modestly differentially expressed in cluster 14: phenoloxidase-activating factor 2 (log2FC = 1.79), serine protease gd-like (log2FC = 2.13), serine protease gd (log2FC = 3.62) and clip domain serine protease 11 precursor (log2FC = 3.08). Beta-1,3-glucan recognition protein is also a DEG in cluster 5 (log2FC = 0.92), cluster 6 (log2FC = 1.55) and cluster 14 (log2FC = 1.35).

### 4.11 Cluster 16 (“Oenocytoid”)

Cells of cluster 16, which were distinguished as oenocytoids (24), have DGEs with relatively high values: two DGEs with log2FC > 5.0; six DGEs with 4.0 < log2FC < 5.0; and nineteen DGEs with 3.0 < log2FC < 4.0 (total number of DGEs = 366) (**Table 12**).

**Table 12:** Marker genes of cluster 16 (“oenocytoid”).

Gene ID	Gene Name	Description	Log2FC	Indicated Process
BMSK0003004	CRYA B	alpha-crystallin B chain-like precursor	5.50	(anti-oxidant) stress response
BMSK0015767	SERPINE2	serpin-7	5.25	melanization immune activation
BMSK0012537	–	27 kDa glycoprotein precursor	4.81	secreted glycoprotein
BMSK0011086	–	carboxypeptidase B-like precursor	4.75	coagulation? tissue repair?
BMSK0015576	DHCR24	delta(24)-sterol reductase-like isoform X1	4.74	lipid homeostasis stress response

The top-ranked marker gene in this cluster encodes α-crystallin B, which is related to an abundant constituent in the lens of the eyes of vertebrates but functions as a chaperone (sHSP) in other tissues in other animals (191). As mentioned before, other sHSPs, located in two clusters on chromosome 27, are considered as marker genes for cluster 5. However, α-crystallin B is located on chromosome 5, as well as the sHSP12.2 gene, which is also up-regulated in cluster 16 (log2FC = 2.55), together with all the sHSP genes from chromosome 27 (see discussion regarding sHSP genes in cluster 5). Thus, in both oenocytid-like clusters 5 and 16, sHSPs are important DEGs although different sHSPs are preferentially involved: sHSPs on chromosome 27 dominate in cluster 5 while the sHSPs on chromosome 5 are more important in cluster 16.

Increased expression of sHSPs is related to the stress response (192, 193). While data in insects are limited, an increase in expression of sHSPs is observed in hemocytes and other tissues following pathogen infection in other invertebrates (194, 195).

The second highly up-regulated marker gene encodes serpin-7 that belongs to a large family of inhibitors of serine proteases called serpins (196). Serpins are referred to as “suicide inhibitors” because of their mechanism of action of irreversible trapping of the protease in a covalent complex (197). Thirty-four serpin genes have been identified in *B. mori* (198) and serpin-7 is similar to serpin-9 of *Manduca sexta* and *Helicoverpa armigera* (56-57% identity) that regulate serine proteases during the immune response (199 200). Interestingly, serpin-9 becomes induced during baculovirus infection in *Helicoverpa* to inhibit melanization and virus inactivation (200).

A glycoprotein of 27 kDa, corresponding to the third marker gene, was first described in the hemolymph of *Manduca sexta* (201). The glycoprotein is insect-specific and carries a domain of unknown function (DUF-1397). Also produced and secreted by the fat body, its concentration in the hemolymph ranges from 10 to 20 μg/mL, without showing obvious changes during development or between sexes (201). Interestingly, proteins with DUF-1397 are moderately (1.7 to 2.1-fold) upregulated after Flock house virus infection in *Drosophila* and after O’nyong–nyong virus infection in *Anopheles gambiae* (202, 203). A protein with DUF-1397 also appears in the venom of a centipede, possibly reflecting its recruitment from the hemolymph (204). Finally, HHpred analysis shows remote similarity with the chondroitin peptidoglycan 4 domain of the nematode *C. elegans* (205).

Carboxypeptidase B-like can be tracked to the gene *BmMCP18* that encodes a metallocarboxypeptidase with Peptidase_M14 domain and a divalent metal (zinc) cation in its active site (206). In mammals, carboxypeptidase B can modulate the coagulation cascade in the plasma (207) but in insects it remains unknown whether a protease cascade is involved in the clotting process (208). In the crab, *Scylla paramamosain*, carboxypeptidase B expression in hemocytes is decreased following infection with White spot syndrome virus (WSSV) and the bacterium *Vibrio alginolyticus* (209). However, carboxypeptidase B may play both a positive and negative role in the immune response since its knockdown decreased WSSV replication while it increased mortality after bacterial challenge (209). In the mosquito *Aedes aegypti*, metallocarboxypeptidase B1 (expressed in the midgut epithelium) has antiviral activity against Dengue virus (210).

Another marker gene encodes the enzyme 24-dehydrocholesterol reductase (DHCR24) that catalyzes the last step in cholesterol biosynthesis (211). In phytophagous insects, this step corresponds to the final reaction in the conversion of plant sterols into cholesterol (212) since insects cannot synthesize sterol compounds *de novo* (213). High expression of DHCR24 is observed in the midgut of *B. mori* (211, 212). Interestingly, DHCR24 has acquired a central role in lipid metabolism in mammals and has been linked to virus infection and oxidative stress (214). Inhibition of DHCR24 in macrophages results in an anti-inflammatory phenotype that is linked to the accumulation of desmosterol (the immediate precursor to cholesterol) and the activation of the nuclear receptor LXR (215). The high expression of DHCR24 therefore indicates the importance of lipid homeostasis and invites further experimentation to investigate the role of DHCR24 in the regulation of oxidative stress and inflammation response by oenocytoid-like cluster 16 cells.

Serpin-7, 27 kDa glycoprotein, carboxypeptidase B and DHCR24 are also expressed in the other oenocytoid-like clusters 5, 8, 12 and 16 but with lower log2FC (1.18 – 3.22) and DEG rank (17 – 1420). The differential expression of sHSP genes was already discussed above and with respect to cluster 5. Regarding other serpins, serpin-4A (related to *Drosophila* spn77Ba) is also found as DEG in clusters 0, 4, 7, 10, 14 and 15 (log2FC = 0.47 – 2.19; DEG rank = 131 – 1156). In *M. sexta*, serpin-4 associates with serine proteases of the prophenoloxidase pathway (216). Serpin-12, which is not orthologous to serpins from other species (198), is highly induced in cells of cluster 15 (log2FC = 4.97; DEG rank = 44).

### 4.12 Cluster 17 (“Granulocyte”)

In the cells of cluster 17, the 5 genes that have increased expression with log2FC > 2 were selected as marker genes (**Table 13**). The 5 marker genes have relatively low DEG rank (47, 3, 46, 119 and 10, respectively, according to the list of genes in **Table 13**).

**Table 13:** Marker genes of cluster 17 (“granulocyte”).

Gene ID	Gene Name	Description	Log2FC	Indicated Process
BMSK0008573	–	beta-1,4-glucuronyltransferase 1	2.60	tissue repair
BMSK0014899	Ppn	papilin	2.22	tissue repair
BMSK0015401	CECB1 CECB2	Cecropin family	2.13	antimicrobial peptide tissue repair?
BMSK0005070	–	nuclear factor NF-kappa-B p110 subunit isoform 1	2.04	immune response transcriptional activator
BMSK0010637	Tis11	protein TIS11 isoform X1	2.01	regulator of immune effectors

The first marker gene in this cluster encodes an enzyme that transfers glucuronic acid residues to O-glycan structures in proteins (217). Structural analysis shows that the protein possesses a transmembrane membrane at the N-terminus and that the glucuronyltransferase domain is exposed to the extracellular medium. Addition of glucuronic acid is considered as a relatively rare modification of various substrates compared to glucosidation in insects (218); on the other hand, glucuronylation is a highly prevalent elaboration of O-linked and glycosphingolipid glycans (217). Because of its negative charge, glucuronic acid has been proposed to act as an equivalent of sialic acid in vertebrates that mediates a wide variety of physiological processes (219). Consistently, mutation or knockdown of glucuronyltransferase enzymes in *Drosophila* leads to developmental defects (220, 221). Interestingly, expression of GlcAT-P, one of three glucuronyltransferase enzymes in *Drosophila*, is required in hemocytes for growth of peripheral nerve fibers (220). One of the essential functions of hemocytes is the secretion and production of extracellular matrix (222) and the glucuronyltransferase may have a function in the glycosylation of extracellular matrix components in the basal membrane or during wound repair and tissue remodeling (217, 220).

Papilin corresponds to a large extracellular matrix protein (2064 amino-acids) with multiple domains (thrombospondin type-I, Kunitz proteinase inhibitor, immunoglobulin-like, PLAC and others), of which several are present in many copies (223). In Lepidoptera, the papilin homolog is known as lacunin (224) and was identified as BmSPI58 in *B, mori* (196). Interestingly, in *Manduca sexta*, lacunin is expressed by granular hemocytes during remodeling of the basal lamina of tissues during metamorphosis (225). Migrating hemocytes are a major source for the production and secretion of papilin during development prior to the formation of basal membranes around tissues (226). In *Drosophila*, papilin is also proposed to play a role in the innate immune response when hemocytes synthesize a temporary extracellular matrix as a scaffold to aid the defense reaction (227). During *Autographa californica* multiple nucleopolyhedrovirus (AcMNPV) infection, papilin expression was suppressed in hemocytes of both *Spodoptera frugiperda* and *Trichoplusia ni* hosts (228). It can be speculated that papilin is a target for the glucuronyltransferase mentioned above because of its heavy glycosylation with O-glycans that include glucuronic acid (229).

Cluster 17 shares with cluster 10 the DGE that encodes Cecropin B albeit with lower log2FC (2.13 versus 3.04). Another cecropin B gene, that belongs to the same chromosomal location (see discussion in cluster 10) is also weakly induced (log2FC = 1.15).

Increased expression of Cecropin B may be related to the identification of Relish as a DEG in cluster 17. Relish is a transcription factor of the NF-κB family and that resemble the mammalian Nfkb1 and Nfkb2 proteins p105 and p100 (230). In *Drosophila*, infection by Gram-negative bacteria triggers the activation of the Imd pathway which leads to the proteolytic cleavage of Relish that is mediated by the caspase Dredd and depends on phosphorylation by the IκB kinase complex (231). In the silkworm, BmNPV infection triggers the activation of BmSTING that promotes the activation of BmRelish and the induction of AMP genes, including *CecB* and *CecA* (232). It is noted that BmRelish is also a DEG in several other hemocyte clusters with increased expression of *CecB* (clusters 0, 4 and 10, but not cluster 7).

Tis11 (TPA-inducible sequence 11) is a member of the tristetraprolin family of CCCH tandem zinc finger proteins that mediate posttranscriptional repression of mRNAs through interaction with AU-rich elements in their 3’-UTRs (233). In the *Drosophila* hemocyte-like SL2 cell line, *CecA1* mRNA is a well-characterized target of TIS11 that promotes its de-adenylation and rapid degradation (234). TIS11 could have a role in the recovery phase of the immune response when mRNAs of effector molecules such as AMPs become repressed and the basal status of the hemocytes become re-established (235). The appearance of both Relish and TIS11 as DEGs in cluster 17 underlines the importance of tight regulation of immune effector molecules at both the transcriptional and posttranscriptional level.

Clusters 0, 4, 7, 10 and 17 are all clusters with increased expression of *CecB* genes. Strikingly, also papilin (clusters 0, 4, 7 and 17), TIS11 (clusters 0, 4, 7, 10 and 17), Relish (0, 4, 10 and 17) and glucuronyltransferase (clusters 4, 10 and 17) are DEGs in most, but not all, of these clusters (albeit with low to moderate log2FC and DEG rank). This coincidence indicates relatedness among these clusters that are qualified as granulocytes. Papilin and TIS11 are also low-ranking DEGs in granulocyte-like cluster 8; in addition, glucuronyltransferase has low log2FC in cluster 19, which was proposed to represent the spherulocyte subtype (24).

### 4.13 Cluster 19 (“Spherulocyte”)

Cells of cluster 19 have many DEGs with high log2FC: 6 genes have log2FC higher than 10; 17 DEGs show log2FC between 5 and 10; and another 28 genes are induced with a log2FC between 3 and 5. High numbers of DEGs and high log2FC indicate that the cells are highly differentiated; in conjunction with their low abundance (0.2% of the total) it can be assumed they may represent a hemocyte subtype with a highly specialized function. All marker genes have log2FC higher than 11 (**Table 14**).

**Table 14:** Marker genes of cluster 19 (“spherulocyte”).

Gene ID	Gene Name	Description	Log2FC	Indicated Process
BMSK0012288	–	MBF2	13.23	secreted factor
BMSK0011592	Ndufb9	DH dehydrogenase [ubiquinone] 1 beta subcomplex subunit 9-like	13.17	mitochondrial function respiration
BMSK0005696	CTSH	cathepsin L like protein precursor	13.15	tissue repair
BMSK0000801	–	chorion class CA protein ERA.2-like isoform X1	12.49	tissue repair
BMSK0012312	–	collagenase	11.63	tissue repair

Multi-bridge factor 2 (MBF2) was originally identified as a transcriptional co-activator that connects the nuclear receptor BmFTZ-F1 with the basal transcription machinery to stimulate transcription of the *fushi tarazu* gene in *in vitro* transcription assays (236). More recently, MBF2 was shown to belong to a family of insect-specific factors that are closely related to the *response to pathogens* (REPAT) genes (237). Two MBF2 genes are highly induced in cluster 19 with log2FCs of 13.23 (rank 1; MBF2-3) and 11.14 (rank 6; MBF2-2). Both genes, together with MBF2-1, are closely linked on chromosome 21 and share sequence identity of 42% (237). MBF2-2 and MBF2-3 are preferentially expressed in the hemolymph and during the larval stages. The expression of MBF2-like genes is induced after bacterial and baculovirus infection and during starvation, indicating functional roles in pathogen defense and nutrient metabolism (237, 238). MBF2 proteins have a small size (100-118 amino-acids), are predicted to be glycosylated and contain a signal peptide which implies that they may be secreted.

The second marker gene emphasizes the role of the respiratory complex in the mitochondria to contribute to the phenotype of cluster 19 hemocytes. NADH:ubiquinone oxidoreductase subunit B9 (NUDFB9) is an accessory subunit of the NADH dehydrogenase complex in the inner mitochondrial membrane that contributes to the oligomerization of different respiratory chain complexes into supercomplexes (239). NUDFB9 therefore may be an important factor to regulate mitochondrial function. Mitochondria can regulate the function of hemocytes in different ways that include the activation of the immune response and the production of ROS (240). Further research is required to determine which hemocyte functions are affected by the increased expression of NUDFB9.

As already discussed for cathepsin B, that is the top marker gene for cluster 7 hemocytes (see above), the high induction of cathepsin L in cluster 19 also suggests a role in tissue remodeling (e.g. extracellular matrix degradation) and the innate immune response. Recent studies indeed have confirmed high expression of cathepsin L in the hemocytes of *B. mori* that could be associated with a role in innate immunity (241).

Collagenase (5^th^ marker) could function in conjunction with cathepsin L as a tissue remodeler or in wound healing. Degradation of collagen is associated with the regulation of growth and shape of organs (242, 243). Because the collagenase has a transmembrane domain and therefore is exposed on the surface, hemocytes are expected to make close contacts with the basal membranes of epithelial cells in which collagen is the major constituent (244).

Interestingly, one of the genes with the highest differential expression in cluster 19 encodes one of the early class “CA” chorion proteins, which were thought to be exclusively expressed in the follicular epithelium of the ovary and are constituents of the eggshell (245). Early chorion proteins are proposed to form an initial scaffold during the first steps of chorion assembly and their expression in cluster 19 hemocytes may reflect a similar role in the assembly of another protective layer, the cuticle. In this respect, it should be remembered that the control group in this study was treated with PBS through injection and that the small group of cluster 19 (50 cells) therefore is involved in wound repair, as is also predicted by the markers cathepsin L and collagenase.

Of the top marker genes in cluster 19, only MBF2-2 can be found as a DEG in another hemocyte subtype, i.e. cluster 14 with moderate log2FC (1.78) and ranking (97).

## 5 Discussion

By analyzing the possible function of predicted marker genes, an attempt was made to construct the phenotype of the hemocyte subtypes/clusters that are represented by them. Previously, our analysis indicated that different clusters could be allocated to the broad classes of hemocytes that prevail in the literature (24). Our current examination of the marker genes in detail largely confirms the previous assessment, especially with respect to the oenocytoid (clusters 5, 8, 12 and 16) and plasmatocyte (clusters 14 and 15) subtypes.

### 5.1 Assignment of Hemocyte Clusters in *Bombyx* as Granulocytes, Oenocytoids, Plasmatocytes and Spherulocytes

The four oenocytoid clusters are characterized by the expression of the prophenoloxidase enzymes that catalyze the melanization process (246). In *Drosophila*, the corresponding cell type is called the crystal cell of which the differentiation is controlled by the Runt-related transcription factor Lozenge (247). A role for Lozenge was also proposed for the differentiation of oenocytoids in *Bombyx* (141). However, in our analysis, Lozenge did not appear as a DEG in the oenocytoid-like cell clusters while Runt-related transcription factor 3 is induced in clusters 5, 12 and 16. These results invite for a re-evaluation of the type of Runt-related transcription factor that is involved in the specification of the oenocytoid lineage in the silkworm.

Beyond the common expression of the prophenoloxidase enzymes, marker genes allow the identification of oenocytoid cells with specific functions (**Table 1**). In cluster 5, the expression of small heat-shock proteins is predominant, which may reflect the preparedness for future possible harmful effector functions and the protection against oxidative stress; this subtype therefore may be only partially differentiated, which is consistent with the relatively low log2FC of its DEGs. In cluster 8, the emphasis is on the expression of putative pattern recognition genes that belong to the class of the 30K proteins. However, more research is needed regarding the differential binding specificity of the different 30K proteins and which pathogen-associated antigens are preferentially recognized. Cells of cluster 12 seem to be more specialized and may have a role in tissue repair with a migrating phenotype; also in this cell type induced expression of apoptosis and stress regulatory genes can be considered necessary for survival in more hazardous environments. Also cluster 16 is highly differentiated and marker genes indicate their contribution to extracellular protease cascades that have important homeostatic functions such as coagulation and melanization. Similarly to clusters 5 and 8, induction of the stress response is observed.

The two clusters (14 and 15) that are marked as plasmatocytes both represent very specialized cell types (marker genes are characterized by high log2FC). Their main functions are related to encapsulation and melanization and the subtypes therefore are reminiscent of the lamellocytes observed in *Drosophila*. Of both subtypes, cells of cluster 14 may have a more regulatory role because of high expression of proteins that regulate the hormone response and may provide protection against oxidative stress (**Table 1**).

Granulocytes are represented by six clusters and therefore cover a wider range of possible functions (**Table 1**). Interestingly, the marker genes in two clusters are clearly associated with cell division and proliferation, such as mitotic spindle formation (cluster 4) and protein synthesis (cluster 6).

In general, cell clusters that are grouped as granulocytes have marker genes with relatively low log2FC (**Table 1**), which indicate intermediate cell types that may acquire more particular effector functions in specific conditions or challenges. Although the function of phagocytosis regularly is associated with granulocytes (248), only in cluster 0 marker genes that are associated with phagocytosis are indicated. However, cluster 0 is the most abundant cluster classified as granulocyte (3145 cells or 37% among all granulocyte clusters), confirming the important role of granulocytes as dedicated phagocytes.

The remaining granulocyte clusters indicate a function in tissue repair. In cluster 7, one protease, one protease inhibitor and one esterase are indicated as marker genes; a zinc transporter protein may be required to support the function of metalloproteinases while also mitochondrial function is activated. Interestingly, in both clusters 10 and 17 cecropin B genes appear to be induced. However, the induction level of cecropin genes is lower for cluster 17 and increased cecropin B expression is noted in 5 out of 6 clusters (clusters 0, 4, 7, 10, 17) designated as granulocytes. Thus, cecropin B expression appears to be a feature of the majority of granulocytes. Although cecropins are known as AMPs, a role in the processing of cytokines for tissue repair and the regulation of melanization has recently also been proposed (119). In cluster 17, cecropin B expression may be regulated at both transcriptional and posttranscriptional level, as indicated by the induction of the transcription factor Relish and the zinc finger protein Tis11.

Spherulocytes have surfaced as an enigmatic hemocyte subtype with large inclusions (“spherules”) in lepidopteran insects for which no function could be designated (8). In our analysis, cluster 19 that consists of only 50 cells was tentatively identified as spherulocytes based on the preferential expression of cathepsin L (16, 24). Further inspection of the marker genes indicates highly specialized effector functions directed to tissue repair. A role for spherulocytes in the synthesis of extracellular matrix and cuticle has been suggested before in the literature (249, 250). Interestingly, one of the marker genes encodes an early chorion protein, of which the expression was thought to be restricted to the epithelium of ovarian follicles (251). Early chorion proteins provide an initial scaffold on which the layers of the lamellar chorion are deposited (252). By analogy, it can be imagined that wound repair is initiated by the deposition of a framework of early chorion proteins on which layers of cuticle proteins can be assembled (253).

### 5.2 Comparison of Hemocyte Subtypes Between *B. mori* and *D. melanogaster*

When looking at the graphical presentation of the different hemocyte clusters in *Bombyx*, the clusters that represent oenocytoids and granulocytes remain associated while plasmatocytes and spherulocytes are clearly separated (**Figure 1**; UMAP plot). At one end of this alignment, cluster 5 (oenocytoids) borders with cluster 6 (granulocytes) and at the other end, cluster 7 (granulocytes) touches cluster 8 (oenocytoids). Linked with cluster 8 are the oenocytoid clusters that are more differentiated (cluster 12 and 16; higher log2FC values) (**Figure 1**). In pseudo-time trajectory construction, granulocytes and spherulocytes are grouped together and separated from oenocytoids while plasmatocytes are located at the intersection of both major groups (24). However, (baculovirus-infected) “pro-hemocytes” for which no marker genes were available were used as a starting point in this analysis. Nevertheless, the UMAP plot in **Figure 1** suggests that particular clusters of oenocytoids and granulocytes are rather closely related and could function as precursors for the differentiation of plasmatocytes (clusters 14 and 15) which are more terminally differentiated. Spherulocytes, on the other hand, represent a small population which also seems to be related to both granulocytes and oenocyotoids (**Figure 1**).

By comparison, scRNA-seq clusters that represent crystal cells in *Drosophila* are more isolated in principal component analysis and, in addition, represent a much lower proportion of the total hemocyte population (5% in *Drosophila* versus 28% in *Bombyx*; **Supplementary Table 1**; 4, 5). This indicates that crystal cells in *Drosophila* correspond to a much more differentiated cell type than oenocytoids in *Bombyx*, where several clusters have marker genes with relatively log2FC and are closely associated with clusters designated as granulocytes (**Table 1**; **Figure 1**). In addition, lamellocytes represent a specialized cell type in *Drosophila* that appears as an immune response following wasp infestation following trans-differentiation from plasmatocytes (4, 5) while in *Bombyx* plasmatocytes (as functional equivalents of lamellocytes in *Drosophila*; 8) have features in common with both oenocytoids and granulocytes (pseudo-time trajectory analysis; 24).

The marker genes that are considered for the classification of hemocytes in *Bombyx* show little overlap with the genes that are considered important to identify hemocyte clusters in *Drosophila* although the analysis proposed the same broad categories (with the exception of spherulocytes that are considered Lepidoptera-specific). Categorization in *Drosophila* was indeed guided by the already accumulated in-depth knowledge following extensive genetic analysis of hemocyte function. However, considerable differences with *Drosophila* were also observed when single cell technologies, including scRNA-seq, were applied to hemocytes from malaria mosquito *Anopheles gambiae* (254–256). This may be expected since processes such as innate immune response and tissue repair undergo fast evolution because of the divergence of different lifestyles among insects.

To illustrate the differences between the two approaches (scRNA-seq guided by accumulated genetic analysis in *Drosophila* versus scRNA-seq guided by morphological/biochemical analysis in *Bombyx*), the differential expression of marker genes characteristic for plasmatocytes, lamellocytes and crystal cells in *Drosophila* (*NimC1*, *atilla* and *lozenge*, respectively; 5) was checked in the scRNA-seq-based hemocyte clusters of *Bombyx*. With respect to oenocytoids/crystal cells, the absence of *lozenge* as a DEG in oenocytoids of *Bombyx* was already mentioned together with the observation of the enrichment of Runt-related transcription factor 3 which is related to Lozenge. With respect to plasmatocytes/lamellocytes, a gene related to *quiver* was specifically up-regulated in clusters 14 and 15 in *Bombyx*, (cluster 14: log2FC=4.39, rank=19; cluster 15: log2FC=3.82, rank=74). Both Atilla and Quiver are glycosylphosphatidylinositol (GPI)-anchored proteins that are involved in the regulation of voltage-gated potassium channels (257, 258) and its expression pattern reinforces the grouping of clusters 14 and 15 as plasmatocytes.

While *NimC1* (encoding a phagocytosis receptor; 259) was not found as a DEG in the scRNA-seq analysis of *Bombyx* hemocytes, the presence of other scavenger receptors involved in phagocytosis was also checked. Granulocyte-like cells preferentially express class B scavenger receptor ScarB1 and class C scavenger receptor Malrd1 with the highest levels in cluster 7 (log2FC of 1.79 and 2.43, respectively). In the oenocytoid-like clusters 12 and 16, the class B receptor Croquemort is more highly induced (log2FC of 4.12 and 3.49, respectively). Plasmatocyte-like cells are enriched in transmembrane serine protease 2 (TMPRSS2) which has a scavenger receptor cysteine-rich domain (260; cluster 14: log2FC=4.82; cluster 15: log2FC=4.48). Thus, while granulocyte-like clusters preferentially express particular scavenger receptors, their induction level remains relatively low compared to other scavenger receptors expressed in the putative oenocytoid and plasmatocyte cells. In *Bombyx*, oenocytoids are usually not considered as phagocytic while both plasmatocytes and granulocytes are engaged in the recognition and phagocytosis of foreign particles (261). The preferential expression of Croquemort in oenocytoids in *Bombyx* therefore is unexpected and may reflect the less differentiated nature (and closer association with granulocytes) of *Bombyx* oenocytoids. Low levels of phagocytosis have been reported in oenocytoids of the mosquito *Aedes aegypti* (262). In *Drosophila*, Croquemort is enriched in plasmatocytes, more specifically in a subtype characterized as “phagocytic plasmatocytes” (4, 263; see also further below).

Recently, a consensus of hematocyte subtypes in *Drosophila* was proposed (263) based on the integration of different scRNA-seq studies (4, 5, 264). An attempt was made to correlate the clusters identified in *Bombyx* with the subtypes that were proposed in *Drosophila* (**Supplementary Table 1**). With respect to *Drosophila* plasmatocytes, which are considered to be the equivalent of *Bombyx* granulocytes (8), different smaller subgroups were proposed based on putative functional properties inferred by predicted marker genes. Strikingly, the major group of plasmatocytes in *Drosophila* (~60%) is designated as “unidentified” since no distinctive markers were identified. Similarly, clusters with marker genes that have relatively low log2FC values in *Bombyx* (arbitrarily set at lower than 3.5; clusters 0, 5, 6, 7, 8 and 17) could also be considered as little differentiated and therefore characterized as “unidentified” (amounting to a proportion of ~65%, which is similar as in *Drosophila*) (**Supplementary Table 1**). However, it was also attempted to correlate the clusters with relatively low log2FC markers in *Bombyx* with the more specialized hemocyte subgroups in *Drosophila* (both scenarios are incorporated in **Supplementary Table 1**).

Interestingly, “proliferative plasmatocytes” were indicated as a subgroup in *Drosophila* (263) which seems to be equivalent with clusters 4 and 6 in *Bombyx* that are also enriched in markers associated with proliferation and growth. It is also noted that 4 out of 5 marker genes in cluster 6 are also DGEs in cluster 5 (marked as oenocytoids; see also section 4.4), consistent with the close association in the UMAP plot (**Figure 1**).

On the other hand, other subgroups that were proposed in *Drosophila* such as “antimicrobial plasmatocytes”, “phagocytic plasmatocytes” and “secretory plasmatocytes” are less clearly represented as categories in *Bombyx* granulocytes. Regarding antimicrobial plasmatocytes in *Drosophila*, a variety of AMP genes is enriched in two subclusters (263) while in *Bombyx* granulocytes preferential expression is limited to cecropin B genes (**Supplementary Table 1**). On the other hand, a common theme may be the enrichment for the Imd pathway (represented by the marker gene Relish in cluster 17). Although cluster 0 in *Bombyx* displays some marker genes with a regulatory function associated with phagocytosis, phagocytic plasmatocytes in *Drosophila* show enhanced expression of effector genes directly involved with the process (263). Cluster 7 of *Bombyx* was also tentatively assigned as phagocytic (although a role in tissue repair was also considered, see above) because marker genes are associated with metalloproteinase function, but further research is required. Secretory plasmatocytes in *Drosophila* produce storage proteins that are also expressed in the fat body (263) while equivalent storage proteins such as 30K proteins are actually differentially expressed by oenocytoid-like cells (cluster 8) in *Bombyx* (**Supplementary Table 1**). 30K markers are also DEGs (with low log2FC; <1) in cluster 7 (granulocyte) which neighbors cluster 8 in the UMAP plot (**Figure 1**).

To complete the comparison, it is also clear that different marker genes are selected with respect to oenocytoids/crystal cells and plasmatocytes/lamellocytes in *Bombyx*/*Drosophila* although the general features of the hemocyte subtype look similar (**Supplementary Table 1**).

## 6 Conclusion

In this study, an overview and extended analysis was provided of the marker genes that were proposed for the characterization of 13 scRNA-seq clusters of hemocytes in the larvae of the silkworm (24). Because of the analysis, marker genes have now lost their anonymity and in many instances clear hypotheses can be formulated to carry out functional studies. Indeed, some marker genes stand out for their interesting features that inspire straightforward experimentation to investigate their hypothesized functions. Examples include a decoy receptor for insulin-like peptides (BMSK0010812; cluster 14), integrin αIIb that may have a conserved function in encapsulation (BMSK0005347; cluster 14) and a secreted protein with C-type lectin-like domain that could function as a decoy against baculovirus infection (BMSK0010425; cluster 15). The indicated proteins are expressed by highly differentiated plasmatocytes and therefore may be associated with specialized immune and/or repair functions.

## Data Availability Statement

The datasets presented in this study can be found in online repositories. The names of the repository/repositories and accession number(s) can be found below: https://www.ncbi.nlm.nih.gov/bioproject/?term=PRJNA658439.

## Author Contributions

LS and MF conceived the idea and designed the study. LS wrote the first draft of the manuscript. MF and JS critically read the manuscript and made improvements in the text and the tables. All authors contributed to the article and approved the submitted version.

## Conflict of Interest

The authors declare that the research was conducted in the absence of any commercial or financial relationships that could be construed as a potential conflict of interest.

## Publisher’s Note

All claims expressed in this article are solely those of the authors and do not necessarily represent those of their affiliated organizations, or those of the publisher, the editors and the reviewers. Any product that may be evaluated in this article, or claim that may be made by its manufacturer, is not guaranteed or endorsed by the publisher.

## Funding

MF acknowledges support from the South China Agricultural University high-level talent launch project. LS acknowledges support of this work by the project ‘An Open-Access Research Infrastructure of Chemical Biology and Target-Based Screening Technologies for Human and Animal Health, Agriculture and the Environment (OPENSCREEN-GR)’ (MIS 5002691) which is implemented under the Action ‘Reinforcement of the Research and Innovation Infrastructure’, funded by the Operational Programme ‘Competitiveness, Entrepreneurship and Innovation’ (NSRF 2014-2020) and co-financed by Greece and the European Union (European Regional Development Fund). JS acknowledges support from the National Natural Science Foundation of China (31872426) and the Guangdong Provincial Promotion Project on Preservation and Utilization of Local Breed of Livestock and Poultry (No.2018-143).
