# Supplementary material for: Hemocyte Clusters Defined by scRNA-Seq in Bombyx mori: In Silico Analysis of Predicted Marker Genes and Implications for Potential Functional Roles
Source: Front Immunol. 2022 Feb 25;13:852702. doi: 10.3389/fimmu.2022.852702 (PMC8914287; doi:10.3389/fimmu.2022.852702)
Supplement: Supplementary file 1 [file Table_1.docx]

| **Drosophila subtype** | **Marker genes** | **Bombyx subtype** | **Marker genes** |
| --- | --- | --- | --- |
|  |  |  |  |
| Unspecified plasmatocytes  (~ 60%) | No distinctive markers | Unspecified granulocytes/  oenocytoids  (65%) | Marker genes with log2FC < 3.5 |
|  |  | Cluster 0 (24%) | Intermediary phagocytic granulocyte? |
|  |  | Cluster 5 (12%) | Intermediary differentiated oenocytoid? |
|  |  | Cluster 6 (10%) | Intermediary proliferative granulocyte? |
|  |  | Cluster 7 (10%) | Intermediary phagocytic granulocyte? |
|  |  | Cluster 8 (8%) | Intermediary secretory granulocyte/oenocytoid? |
|  |  | Cluster 17 (1%) | Intermediary antimicrobial granulocyte? |
|  |  |  |  |
| Proliferative plasmatocytes  (~ 3.5-12%) | Cyclin B  String  Pendulin  Polo  (related to mitosis) | Granulocytes  Cluster 4  (12%) | Kinesin-like proteins  Mitotic spindle assembly checkpoint  Stathmin  (related to mitosis) |
|  |  | Granulocytes  Cluster 6  (10%) | Myc  60S ribosome subunit biogenesis protein, Nucleolin  (related to protein synthesis and growth)  (unspecified?, log2FC < 3.5) |
| Antimicrobial plasmatocytes  (~ 0-17.5%) (*) | High expression AMPs  Cecropins, Diptericin,  Drosomycin, Metchnikowin  Matrix metalloproteinase 1 |  |  |
|  | Low expression AMPs  Imd pathway  JNK pathway | Granulocytes  Cluster 10  (8%) | Low expression AMPs  Cecropin B  IMP-L2 |
|  |  | Granulocytes  Cluster 17  (1%) | Low expression AMPs  Cecropin B  Relish  (unspecified?, log2FC < 3.5) |
|  | Ferritin 1 heavy chain  Ferritin 2 light chain,  Multidrug resistance  (xenobiotic metabolism) |  |  |
| Phagocytic plasmatocytes  (~ 6.5-21%) | NimC2 (phagocytic receptor)  Myoblast city (cytoskeleton)  Tenascin (extracellular matrix)  Lipophorin receptor 2  Matrix metalloproteinase 2 | Granulocytes  Cluster 0  (24%) | LPS-induced TNF-alpha homolog  Serotonin receptor  (unspecified?, log2FC < 3.5) |
|  |  | Granulocytes  Cluster 7  (10%) | Metalloproteinase inhibitor  Zinc transporter  (unspecified?, log2FC < 3.5) |
| Secretory plasmatocytes  (~ 7-8%) | Larval serum protein 1α  Larval serum protein 2  Apolipoprotein  Odorant binding protein 99b  (storage proteins) | Oenocytoids  Cluster 8  (9%) | 30K proteins  (storage proteins)  (low levels PPO)  (unspecified?, log2FC < 3.5) |
|  |  |  |  |
| Crystal cells  (~ 0.5-5%) | Low levels PPO  Notch, pebbled, E(spl)m3-HLH  (intermediary) | Oenocytoids  Cluster 5  (12%) | Low levels PPO  Heat-shock proteins  (unspecified?, log2FC < 3.5) |
|  |  | Oenocytoids  Cluster 12  (6%) | Low levels PPO  Cilia- and flagella-associated protein  Trichohyalin  (cytoskeleton) |
|  |  | Oenocytoids  Cluster 16  (1%) | Low levels PPO  Heat-shock proteins  Protease cascade |
|  | High levels PPO  (mature) |  |  |
|  |  |  |  |
| Lamellocytes  (transdifferentiation after wasp infection**)  (~ 1.5-15%) | Low levels Atilla  lncRNA CR44316  (intermediate) | Plasmatocytes  Cluster 14  (5%) | Integrin alpha-IIb-like precursor  Insulin-like peptide receptor  (regulatory) |
|  | High levels Atilla  betaTub60D, alpha-Tub85E (tubulins)  (mature) | Plasmatocytes  Cluster 15  (2%) | Serine protease snake-like  (clotting/coagulation)  Serine protease 11  (melanization cascade) |
|  |  |  |  |
| PSC-like hemocytes (***)  (<0.5%) | markers of the posterior signaling center (PSC) of the lymph gland |  |  |
|  |  |  |  |
|  |  | Spherulocytes (***)  Cluster 19  (<1%) | Early chorion protein  Collagenase  (tissue repair) |

**Supplementary Table 1:** Possible correlation of hemocyte subtypes between *Drosophila melanogaster* and *Bombyx mori* based on scRNA-seq data. The division of *Drosophila* subtypes is based on the analysis of Cattenoz et al. (2021). Regarding *Bombyx* hemocytes, clusters with low log2FC values (clusters 0, 5, 6, 7, 8 and 17) are considered to be equivalent to the “unspecified plasmatocytes” identified in *Drosophila*. Alternatively, these clusters in *Bombyx* could be associated with categories of more differentiated cell types in *Drosophila* (proliferative, phagocytic, secretory or antimicrobial plasmatocytes and crystal cells). Indicated is also the relative abundance (as % of total hemocyte population). (*) The category of antimicrobial plasmatocytes was not found in the scRNA-seq study of Fu et al. (2021). (**) Lamellocytes are usually identified in *Drosophila* only after parasitoid wasp infestation and develop from plasmatocytes to considerably high levels by transdifferentiation (approximately 15% as estimated from Cattenoz et al. (2020); low levels of lamellocytes were also identified in the study of Fu et al. (2020). (***) PSC-like hemocytes and spherulocytes are unique for larval hemolymph derived from *Drosophila* and *Bombyx*, respectively. Abbreviations: AMP, antimicrobial peptide; Imd, immune defficiency; JNK, Jun N-terminal kinase; PPO, pro-phenoloxidase.
